# Prof. Donders’ Mode of Assisting Vision in Certain Opacities of the Cornea

**Published:** 1859-08

**Authors:** E. L. Holmes

**Affiliations:** Chicago


ARTICLE IV.

### PROF. DONDERS' MODE OF ASSISTING VISION IN CERTAIN OPACITIES OF THE CORNEA.

BY E. L. HOLMES, M. D., OF CHICAGO.

Every person of experience in diseases of the eye must have observed numerous cases in which, after all active disease had disappeared, more or less extensive opacities of the cornea had left the organ in an almost useless condition. Various operations and modes of treatment have been devised to restore vision in such cases, and, it is true, are often applied with advantage. No operation, perhaps, has been more successful than that for artificial pupil; and yet there are instances in which even this operation alone, however skilfully it might be performed, and however prudent the subsequent treatment might be, would fail to produce any beneficial results. The other operations are so seldom practicable, and the treatment by local applications so tedious, that any *simple* means of aiding vision, if it be found suited to even few cases, must be of interest to our profession.

The simple apparatus of Prof. Donders, although it had been applied already in cases of conical cornea, and had long been used by travellers in high latitudes to protect the eyes from the influence of the dazzling snow, had never been tried in certain conditions of the cornea. To the distinguished oculist of Utrecht belongs the credit of first extending its application to these cases, and, with an account of the discovery, publishing an analysis of the principles upon which its efficiency depends. The apparatus, in its simplest form, is nothing more in appearance than a common pair of spectacles, the glasses of which have been exchanged for thin slips of blackened ivory, pierced in the centre with minute apertures.

Since the appearance of Prof. Donders' paper in the *Netherlands Lancet* (of 1854 I think), the leading oculists in Europe have written or lectured more or less upon the subject, and proved the utility of this simple discovery.

The effect produced upon vision by the presence of opacities upon the cornea depends, of course, upon the degree of opacity and their situation.

However paradoxical it may appear, it is nevertheless true, that translucent obscurations of the cornea covering *portions* of pupil, although they admit more light into the eye than denser opacities of the same extent, in reality cause a much greater derangement of vision. The paradox, however, is easily explained away, when we recollect that it is among the conditions upon which distinct vision depends, that as many rays of light from an object as necessary should unite at the macula lutea of the retina, and that as few as possible from any source should be diffused upon the other portions of the nerve. It is from the fact that these conditions are observed that we obtain, under certain circumstances, a much more distinct view of an object by looking at it through a pin hole pierced through a black card, than with the unprotected eye, for the reason that the card excludes the greater portion of rays entering the eye from other objects, which, without the card, being refracted as diffused light upon different portions of the retina, would render the impression of the object less well defined. Now a perfectly opaque leucoma, if it covers nearly the whole of the pupil, leaving a very small part even of the cornea over it, in a normal condition, scarcely affects direct vision, since the leucoma merely reduces the pupil in size; which circumstance, as we can see by looking through a minute aperture in a card, is of comparatively little importance. If a perfectly opaque but minute leucoma is situated over the centre of the pupil, leaving an annular portion of it open to the rays of light, or if it lies in the form of a long, narrow cicatrix over the centre of the cornea, leaving a small section of the pupil clear each side of it, vision still remains almost wholly unaffected.

We can convince ourselves of the truth of these latter statements by holding a minute disk of black paper, much smaller than the pupil, over the centre of the cornea and *as near to it as possible*, when it will be observed that the disk disappears, while vision is not obscured. The same will be the result if a small black probe be held horizontally or perpendicularly in the same manner before the centre of the pupil. A double pupil, either congenital or accidental, is scarcely detrimental to distinct vision, when the eye is, in other respects, normal, since rays of light from each point in an object pass through both pupils, and are united at the *same* corresponding point of the macula lutea.

The foregoing remarks, it must be remembered, hold true in cases only in which the obscurations shading portions of the pupil are absolutely opaque. When such obscurations are only semi-transparent, the eye is in a far worse condition. In this case, some of the rays pass through the transparent portion of the cornea, and would form a distinct image of an object placed in front of the eye if all other rays were excluded; but other rays fall upon the nebulous portion of the cornea, which thus becomes luminous, remitting from every point light, which is diffused in all directions in the eye, destroying the outline of the image of the object pictured upon the retina. The effects of this diffused light upon vision may be easily illustrated by a simple experiment. If a small, round aperture be made in the shutter of a darkened room, a bright disk of light, if the sun's rays be favorable, will be formed upon a screen behind the aperture. It is well known that inverted images of objects held in front of the aperture in the shutter will be formed within this disk. Now, if a piece of well ground glass be placed over half of the opening, leaving the other half free, the *room* will be lighter than before, from the light thrown in every direction by the luminous glass, while the images in the disk will be less distinct. Translucent obscurations, which emit *diverging* rays of light and ulcerating portions of the cornea, still remaining quite transparent, which refract light in all directions through the humors of the eye, although they may be quite minute, cause great indistinctness of vision. They act like small lenses placed near the cornea. A small light placed near the eye, as is well known, may be much more dazzling than a larger and more brilliant one at a greater distance.

A simple experiment with one's own eye will also show very satisfactorily the effect of the various kinds of nebulæ situated in front of the pupil. We have already seen that a disk of black paper, much smaller than the pupil, placed over the centre of the cornea and nearly touching it, does not materially diminish vision. Exchange this disk of paper for one of thin glass, and it will be seen at once that the diffused light thus produced will render vision almost impossible. For a similar reason, a patient with some forms of cataract is unable to endure a strong light, although it would naturally be supposed that the clouded lens, inasmuch as it excludes a portion of light which would normally enter the eye, would render it less sensitive to the influence of light. But the lens, not being sufficiently opaque to exclude *all* the rays of light, is only clouded, and consequently becomes an illuminating body, sending diffused light to every portion of the retina. From the fact that the lens is very near the retina, its power of producing photophobia is proportionally great. It is probably for a similar reason that the pupil is usually contracted in certain cases of cataract, when we might expect to find it dilated.

It may not be out of place to allude to the fact that diffused light, however prejudicial it may be to vision under the circumstances just mentioned, is often of great advantage in lighting obscure apartments. It is well known that much more light can be admitted through the decks of a vessel by means of a piece of ground glass than by a much larger transparent piece. A room, situated in a narrow and dark alley, is much better lighted by ground glass windows than by plates of ordinary glass, for the light in the latter case falling directly through the window, illuminates only the small portion of the floor upon which it falls obliquely from above, while the ground glass becomes luminous, diffusing in every direction.

Whenever it is in any case once discovered that diffused light is the chief cause of disturbed vision, we have a sure palliative remedy in the use of the simple apparatus of Prof. Donders. When we reflect how instinctively patients with diseases of the eye, either by the hand or by partially closing the eyes, endeavor as much as possible to exclude all disturbing light, it seems strange that such an apparatus was not long before more extensively applied in such diseases.

The principle upon which it acts is easily explained. The opaque plates before the eyes exclude all unnecessary light, while the minute apertures permit pencils of rays from an object to fall upon the transparent portion of the cornea only, which pencils, of course, are brought to a focus upon the retina, forming a distinct image.

In preparing these spectacles, a few practical points should always be borne in mind. They should be fitted closely to the edges of the orbit in order to exclude all unnecessary light. The aperture should be as near the cornea as possible, and in front of the unclouded portion of the pupil. To do this, it is well that a cone-shaped tube should be fitted to the plate, in such a way that the apex, in which is the opening, should be a couple of lines from the eye. It sometimes requires a little practice on the part of the patient to keep the pupil exactly behind the aperture, but this difficulty is soon overcome. Inasmuch as these spectacles are heavy and somewhat peculiar in appearance, it is often convenient for the patient, who is able to walk the streets without assistance, to provide himself with the apparatus adapted to one eye, to be carried in the same manner as a common eye-glass. This only need be used in reading and examining minute objects.

In cases of albinismus and of mydriasis (dilated pupil), either idiopathic or produced by belladonna, when vision is disturbed by too much light entering the eye, the patient will find much benefit from the use of this apparatus.

The operation for artificial pupil would be advisable in numerous instances, in which, without this simple contrivance, it would be almost entirely unsuccessful, from the presence of a translucent obscuration over a portion of the new pupil.

The apparatus is so simple, experiments are so easily tried, and, under proper circumstances, so sure to be followed with success, that it is useless at present to refer to particular cases in which patients have been benefited by its use. Nothing can be more satisfactory to a physician than to see and hear a patient, who has long been unable to read and discern minute objects, express his gratitude on being furnished with so simple a means of recovering, to a great extent, his lost vision.

